# Simulation Study on the Corrugated Plate Gas–Liquid Separator with the Assistance of the Drainage Hook

**DOI:** 10.1021/acsomega.2c05581

**Published:** 2022-11-22

**Authors:** Chen Zhao, Jigang Zhao, Mei Cong, Haitao Shen

**Affiliations:** International Joint Research Center for Green Energy Chemical Engineering, East China University of Science and Technology, 130 Meilong Road, Shanghai200237, People’s Republic of China

## Abstract

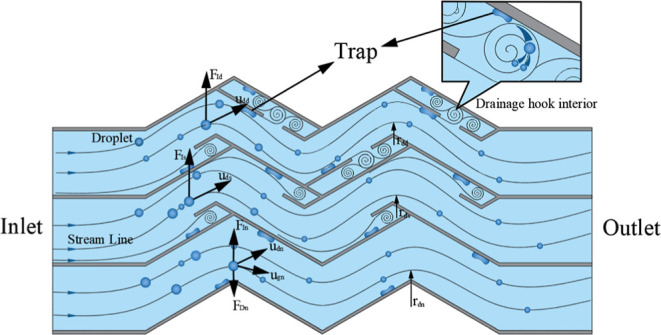

Corrugated plate separators are widely used in the field of gas–liquid separation because of their excellent separation performance. The separation effect is very sensitive to the internal auxiliary structure of drainage hooks, so it is extremely important to study the action principle of drainage hooks to optimize the performance of corrugated plate separators. In this paper, Fluent is used as the solver and the realizable *k*–ε model is used to compare the separation performance of unhooked, single-hooked, and double-hooked corrugated plates. The results show that the separation efficiency of wave plates with hooks can reach 100%, the separation efficiency of wave plates without hooks is about 90%, and the superiority of the separation efficiency of single-hook and double-hook wave plates is related to the droplet partition diameter, which is positively correlated with *Re*. The pressure drop and separation efficiency increase with the increase of plate hook spacing, and the pressure drop and separation efficiency of single-hook and double-hook corrugated plates have different performance advantage zones influenced by *Re* and *K*_a_. When the *Re* is 9.64 × 10^3^ and *K*_a_ is 0.294, the separation effect of corrugated plates with the single hook and double hook is the same. Through the analysis of the gas-phase flow field and droplet motion trajectory, it is found that the drainage hook enhances the separation effect of the corrugated plate separator by increasing the local gas velocity and forming a vortex inside the drainage hook.

## 1 Introduction

Gas–liquid separation is related to the efficient utilization of resources, protection of the environment, and safe operation of the plant. At present, common gas–liquid separation equipment includes the gravity type, inertia type, filtration type, centrifugal type, and so forth.^1^ Different separation methods and typical separators are shown in Table 1. A metal screen type mist eliminator is suitable for the diameter range of 0.1–10 μm.^2^ Cyclone separators have a separation accuracy of 15–30 μm and are widely used in petrochemical, vehicle, and helicopter internal combustion engine air intakes.^3−7^ A corrugated plate gas–liquid separator has the advantages of a compact structure, a high separation efficiency, and a small pressure drop loss. It is widely used in nuclear power plants,^8^ flue gas desulfurization,^9^ and the chemical industry.^10^ In this paper, a corrugated plate gas–liquid separator installed in a tower for industrial separation of methanol is the object of study.

**Table 1:** Comparison of Common Separation Methods^1^

type	equipment name	principle	advantages	disadvantages
gravity type	gravity settling separator	gravity difference	simple structure and low maintenance cost	low separation efficiency
inertial	corrugated plate type	inertia force difference	simple structure, low pressure drop, and high separation efficiency	processing capacity limited by secondary entrainment
filtering type	metal screen	separation by filtration media	high separation efficiency	easy to clog, limited processing capacity
centrifugal	cyclone	centrifugal force difference	high separation efficiency	little operational flexibility and difficult structural design

The corrugated plate gas–liquid separator relies on the difference of inertia forces on the gas and liquid phases for separation. The air flow entrains small droplets into the corrugated plate torsional flow channel at a certain speed. Due to the high density of the liquid phase and the large inertia force, the velocity of the droplets cannot be changed in time and is captured by the wall surface. The liquid film is deposited on the wall surface of the corrugated plate and discharged from the corrugated plate separator by gravity. It is found that the plate spacing, bending angle, length of each stage, and number of stages have significant effects on the performance of corrugated plate gas–liquid separator; the separation efficiency of the corrugated plate with hooks increases by 25.00% due to the presence of drainage hooks, but at the same time, the pressure drop loss increases by 117.1 Pa.^11−14^ Therefore, it is important to study the mechanism of drainage hooks to obtain higher separation performance.

Early studies on corrugated plate gas–liquid separators were mainly experimental. Wang^15,16^ and Li et al.^17−20^ demonstrated that the drainage hooks could significantly enhance the separation efficiency through cold tests, and the pressure drop of a single-hook corrugated plate was the highest in the test range, followed by that of a double-hook corrugated plate. Chen et al.^21,22^ studied the effect of the drainage hook form in detail, and the results showed that the separation effect was better when the plate hook spacing was gradually decreased from the inlet to the outlet, and decreasing the plate spacing led to the deterioration of the separation effect at the same drain hook spacing. Nakao et al.^23,24^ developed a simplified blade based on the force analysis of droplets in the flow field and also on the experimental finding that droplets were separated mainly in the first and second stages of the corrugated plate. The experimental study can accurately respond to the effect of parameters on performance, but the long period, the small number of test points, and the limitations of the test conditions do not allow visual analysis of the flow field distribution and droplet trajectory within the corrugated plate.

In recent years, with the advancement of computer technology, numerical simulation studies of corrugated plate separators have flourished. The researchers have conducted a large number of simulation studies for corrugated plate gas–liquid separators by randomly combining different turbulence models, grid forms, wall treatment methods, and differential formats. By comparing the simulation results with the experimental data, they found that the grid format has little effect on the results, the simulation results in higher-order difference format are more accurate, the simulation of the flow field is more accurate, and the prediction of small droplets is better when the enhanced wall function is used; thus, a mature simulation calculation method is established.^25−27^ Scholars at home and abroad have studied the influence law of the single-hook corrugated plate drainage hook height, length, and angle and the interaction between the three on the separator performance and optimized the structural parameters of the single-hook corrugated plate drainage hook.^28−31^ The researchers have studied different structure types of front and rear drainage hooks for the double-hook corrugated plate, analyzed the advantages of different structures by the effects of drainage hooks on velocity, pressure, turbulent kinetic energy, and separation efficiency, and optimized the design using the response surface method.^32−35^ In the research process, single-hook and double-hook corrugated plates are usually studied separately, the advantages and disadvantages of their performance are determined singularly, and the comparative analysis of the performance of single-hook and double-hook corrugated plates is lacking. Moreover, the numerical simulation study of a corrugated plate with hooks mainly focuses on the optimization design, and the investigation of the action mechanism of drainage hooks is insufficient.

At present, it is generally found that the separation effect of wave plates with hooks is significantly better than that of wave plates without hooks, but the mechanism of action of drainage hooks is not very clear, and there is a lack of comparison of the separation performance of single- and double-hook wave plates. This paper conducts a comparative study on the separation performance of corrugated plates of three configurations, analyzes the main influence mode and action mechanism of drainage hooks, and gives the respective performance advantage range of single- and double-hook corrugated plates to provide reference for the subsequent optimization of the drainage hook structure and selection of corrugated plates.

## 2 Numerical Calculation Method

### 2.1 Purpose and Methodology

In this paper, the main research objective is to investigate the mechanism of action of drainage hooks. The performances of single-hook and double-hook corrugated plates are compared to provide a reference for industrial selection.

Firstl, the separation performances of three configurations of corrugated plates at different velocities are studied to analyze the advantages of corrugated plates with hooks. Second, the separation effects of single-hook and double-hook corrugated plates on droplets of different diameters are investigated. Then, the effect of the plate hook distance on the separation performance at different velocities is investigated. The performance advantage zones of single-hook and double-hook corrugated plates are obtained. Finally, the mechanism of the assisted action of the drainage hook is analyzed in combination with the gas-phase flow field and droplet motion trajectory.

### 2.2 Geometric Modeling and Meshing

**Figure 1:**
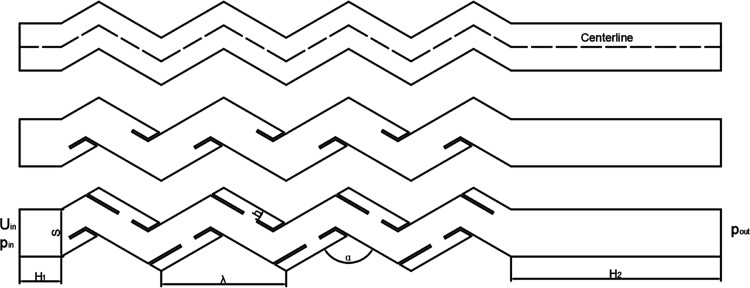
Corrugated plate structure diagram.

The geometric model used in this paper is a simplification of the double-hook corrugated plate gas–liquid separator used for methanol separation in the plant. The single-hook and no-hook corrugated plate models are obtained by removing the drain hooks at different locations on the basis of the original model. The structure is shown in Figure 1, and its detailed geometric parameters are shown in Table 2. The corrugated plate inlet length is 15.90 mm, the plate spacing is 18.00 mm, the length of each stage is 47.60 mm, the plate–hook spacing is 4.20 mm, the bending angle is 120°, and the direction of the drain hooks is parallel to the wall surface. In order to ensure that the outlet section flow is fully developed, the outlet is extended to 80 mm.^34^

**Table 2:** Corrugated Plate Main Structure Parameters

name	value
*H*_1_	15.90 mm
*H*_2_	80.00 mm
*S*	18.00 mm
λ	47.60 mm
α	120°
*h*	4.20 mm

The air–water system was used for the study. The properties of air and water were obtained from the Fluent material library. The air density is 1.225 kg/m^3^, viscosity is 1.79 × 10^–5^ Pa·s, water density is 998.2 kg/m^3^, and viscosity is 1 × 10^–3^ Pa·s. The temperature in the calculation is room temperature. During the study, the corrugated plate gas–liquid separator model is simplified as follows:^36^

(a) Since the height of the corrugated plate is much larger than the width, the corrugated plate is simplified to a two-dimensional model.
(b) A discrete phase model is used, where air is the continuous phase and water droplets are the discrete phase, ignoring secondary entrainment, and the droplets are considered to be trapped when they touch the wall.
(c) The flow process is a steady-state constant flow, and the gas phase is incompressible.
(d) The interaction between droplets is ignored.

**Figure 2:**
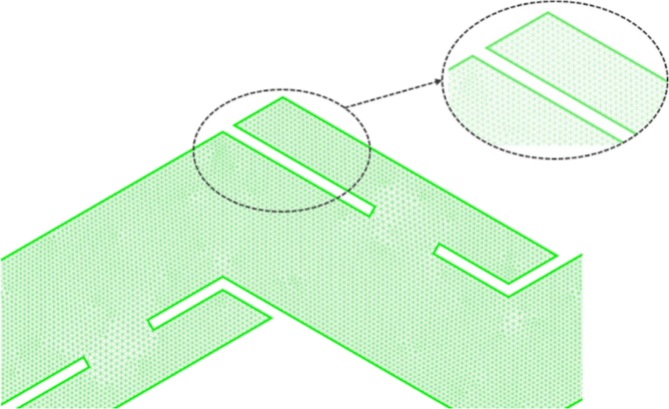
Mesh division results.

**Figure 3:**
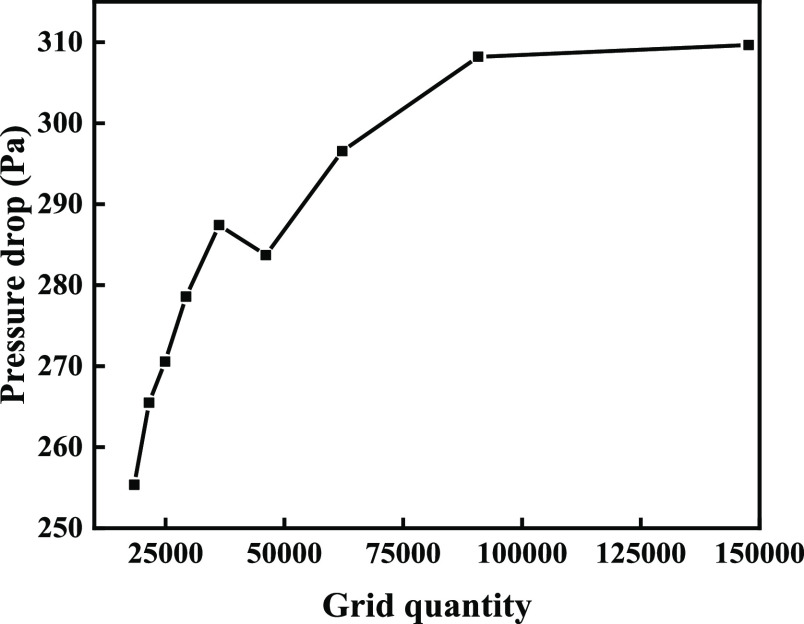
Mesh irrelevance detection.

ICEMCFD is used to mesh the physical model. The mesh type can be divided into structural and nonstructural meshes. The nonstructural mesh does not require any analysis work in the calculation process compared with the structural mesh. At the same time, the nonstructural mesh has better adaptability to the model, and it is easier to generate a high-quality mesh that meets the calculation accuracy, so the nonstructural mesh is chosen for the calculation. The unstructured mesh is divided into quadrilateral and triangular meshes according to the shape. Since there are acute angles in the structure of the corrugated plate, the triangular mesh is better adapted and the calculation results are more accurate, so the triangular mesh is chosen. The solution for the near-wall surface uses the enhanced wall function, which requires encryption of the near-wall mesh to ensure that the near-wall mesh *y*^+^ ≈ 1,^37^ setting the height of the first layer of mesh to 0.01 mm and the number of boundary layers to 10 for meshing and dividing the results as shown in Figure 2. By increasing the number of meshes and excluding the influence of the mesh on the calculation results, the calculation results obtained are shown in Figure 3, indicating that when the number of meshes reaches 90,804, the pressure drop almost does not change.

### 2.3 Numerical Calculation Model

#### 2.3.1 Control Equations

In this paper, the airflow and droplets are in thermal equilibrium. Therefore, the energy exchange is not considered in the calculation process. The gas flow state inside the corrugated plate can be obtained by solving the mass conservation equation and the momentum conservation equation, which can be expressed as the following equations.^38^

Mass conservation equation
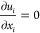
1

Momentum conservation equation
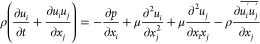
2

The gas flow inside the corrugated plate is turbulent, and the current calculation methods for turbulent flow can be divided into the following: direct numerical simulation, large eddy simulation, and the Reynolds stress-averaged N–S model. The direct numerical simulation is only suitable for low Reynolds number flow, and the large eddy simulation is very demanding on computer resources. The Reynolds stress-averaged N–S model is a solution to the N–S equation, which is computationally small and provides a multi-seed model that is suitable for all turbulent flows. In this paper, we choose the widely used realizable *k*–ε turbulence model in the Reynolds stress-averaged N–S model for calculation,^39,40^ and the equation of the realizable *k*–ε model is^41^
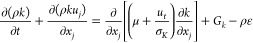
3
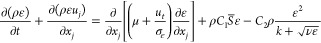
4

#### 2.3.2 Droplet Control Equation

The discrete phase model in Fluent is suitable for applications where the particle volume share is less than 10%, and the corrugated plate separator is used as a secondary separation device where the inlet vapor humidity is usually less than 25%, so the discrete phase model is used to simulate the liquid droplets.

In the calculation, the Eulerian method is used to calculate the continuous phase flow field, and then, the trajectory of the droplet is determined by the Lagrangian method, which traces the position of the droplet at different times with a single droplet. The equation governing the motion of the droplets is as follows (*x*-direction)
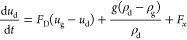
5where *F*_D_ is the drag force per unit mass of the droplet and *F*_D_ can be expressed as
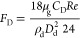
6where *u*_g_ is the velocity of the airflow, given in millimeters per second; *u*_d_ is the velocity of the droplet, given in millimeters per second; ρ_g_ is the density of the airflow, given in kilograms per cubic meter; ρ_d_ is the density of the droplet, given in kilograms per cubic meter; μ_g_ is the viscosity of the gas phase, given in pascal second; and *D*_d_ is the diameter of the droplet, given in meters.

The expression of the traction coefficient *C*_D_ is
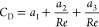
7where *a*_1_, *a*_2_, and *a*_3_ are constants,^42^ where *Re* is the relative Reynolds number and the expression is
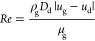
8

In addition, [*g*(ρ_d_ – ρ_g_)]/ρ_d_ in the equation is the gravity term and *F*_*x*_ indicates other additional forces on the droplet, which are not considered in the calculation.^36^

#### 2.3.3 Turbulent Diffusion

When turbulence effects are not considered, Fluent calculates the droplet trajectory with the gas-phase time-averaged velocity. The random walk mobility takes into account the effect of turbulence effects on droplet motion by introducing pulsation velocity
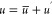
9where *u* denotes the instantaneous velocity, *u̅* denotes the continuous phase velocity, and *u*′*u* denotes the fluctuation of the continuous phase velocity.

The random walk model tracks the droplet by simulating the interaction between the droplet and the vortex, which can be described by the random pulsation velocity degrees *u*′, *v*′, and *w*′ and the time scale τ_e_, assuming that *u*′, *v*′, and *w*′ obey a Gaussian distribution, ζ is a random number obeying normal distribution, when using realizable *k*–ε calculation, and the pulsation velocity considers Reynolds stress score anisotropy, then
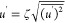
10
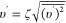
11
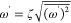
12

The Lagrangian integral time scale of the fluid is defined as
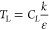
13

The value of *C*_L_ is the eddy current lifetime constant, which has different values in different cases. Estakhrsar and Rafee^43^ studied the effect on the separation effect, using 0.15 by default.

The characteristic survival time of the eddy is defined as
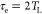
15

The relaxation time of the droplet and the time taken to cross the eddy are defined as
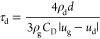
16
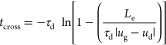
17
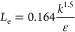
18

*L*_e_ denotes the spatial scale of the eddy. The interaction time of the droplet with the airflow is taken as the smaller of the eddy survival time and the time taken for the droplet to cross the eddy. When the time reaches this smaller time, an instantaneous velocity is obtained again.^13^

### 2.4 Solver Settings

In the calculation process, the boundary conditions are set as follows: the corrugated plate inlet is set to “velocity -inlet”, the outlet is set to “outflow”, and the wall is set to “trap”. For the calculation of the flow field, the simple algorithm, which is better adapted to the complex turbulent flow inside the corrugated plate, is used. In order to obtain good convergence stability, the default sub-relaxation factors of pressure, momentum, *k,* and ε are changed to 0.2, 0.5, 0.5, and 0.5, respectively. The pressure interpolation format is set to PRESTO! The different formats of momentum, turbulent kinetic energy, and turbulent dissipation rate are chosen to be the second-order windward format in order to ensure higher accuracy. To ensure the convergence of the solution and the correctness of the solution, the convergence criteria are set as follows:

(1) monitoring the residuals of the mass flow, momentum, *k,* and ε so that they are always below 10^–5^;
(2) monitoring the wall resistance coefficient to ensure that it no longer varies significantly;
(3) monitoring the average pressure and average velocity at the inlet and outlet to ensure that they no longer vary significantly.

When the above three conditions are satisfied, we can consider that the calculation has converged.

### 2.5 Model Accuracy Validation

**Figure 4:**
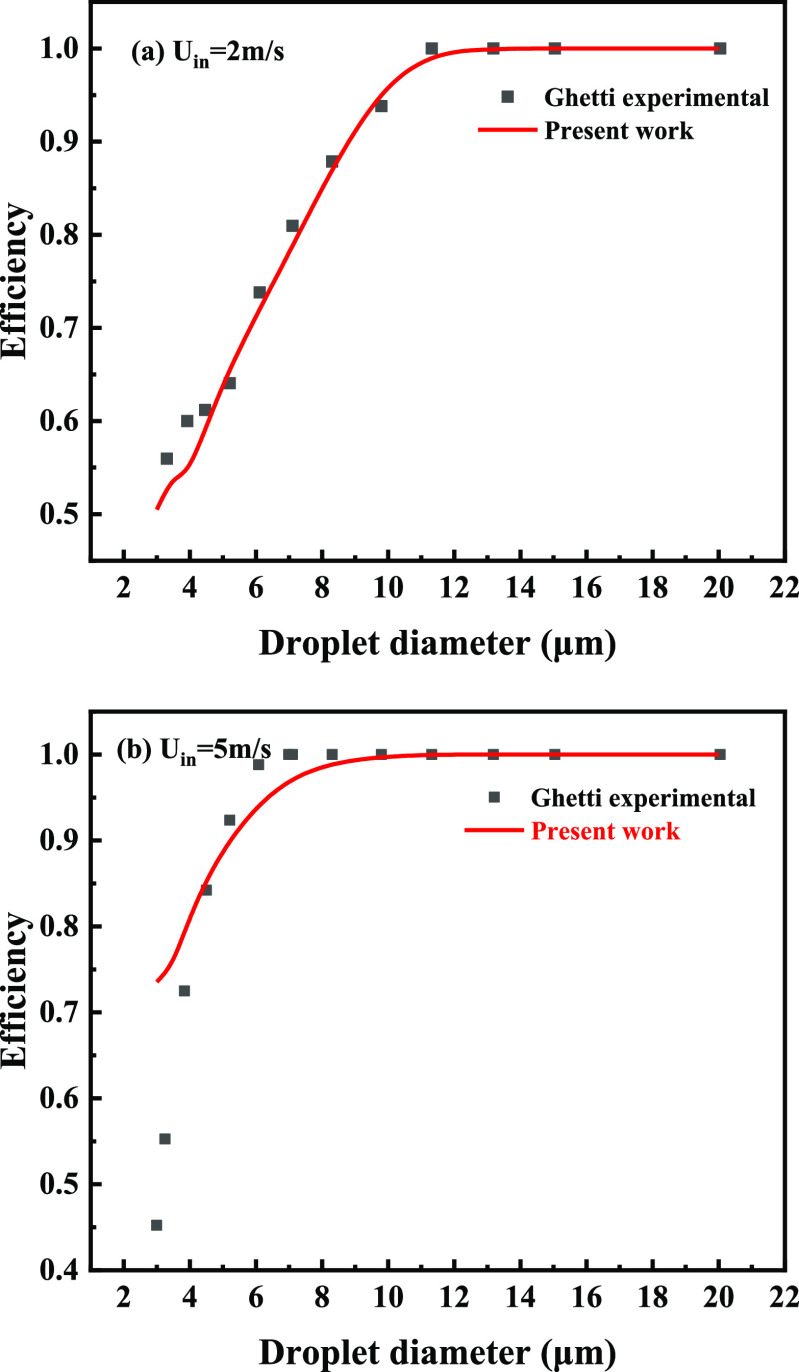
Comparison of separation efficiencies of droplets with different diameters: (a) *U*_in_ = 2 m/s and (b) *U*_in_ = 5 m/s.

In this paper, the effect of drainage hooks on the gas–liquid separation inside the corrugated plate is investigated. The correctness of the simulation method is verified by comparing with Ghetti’s experimental data on single-hook corrugated plates.^44^ The calculation method and geometric model are referred to in the literature.^26^ The comparison of the results at *U*_in_ = 2 m/s and *U*_in_ = 5 m/s was obtained, as shown in Figure 4. The inlet air humidity is set to 10%; therefore, the incident mass of droplets *m*_inject_ is calculated according to the inlet air velocity. Droplets are uniformly injected from the inlet interface and are tracked using a random walk model. The droplet mass trapped by the wall is noted as *m*_trap_, which is obtained by post-processing of the discrete phase in Fluent. The separation efficiency is calculated as follows
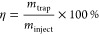
19

The separation efficiencies of droplets with different diameters at 2 and 5 m/s were simulated and calculated, and the results are shown in Figure 4. It can be found that the separation efficiency of droplets with diameters below 5 μm deviates from the experimental data. The separation efficiencies of droplets with diameters larger than 5 μm agree well with the experimental data and are in strong agreement with the experimental results.

## 3 Results and Discussion

The corrugated plate can be divided into a corrugated plate without a hook and a corrugated plate with a hook according to the presence or absence of the drainage hook, and the corrugated plate with hook can be divided into a single-hook corrugated plate and a double-hook corrugated plate. Due to the presence of the drainage hook, the separation ability of the corrugated plate with a hook is stronger than that of the corrugated plate without a hook, but at the same time, it results in a higher pressure drop.

### 3.1 Influence of the Drainage Hook Configuration

**Figure 5:**
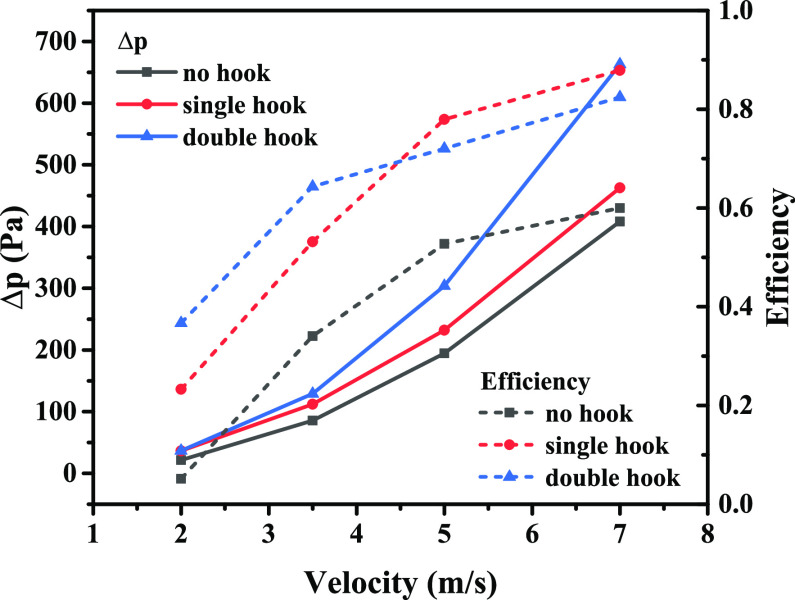
Variation of Δ*p* and efficiency with speed for different plate types.

Figure 5 shows the change of the pressure drop of three types of corrugated plates at different velocities. The pressure drop of the corrugated plate is calculated according to eq 19. *P*_in_ is the inlet static pressure and *p*_out_ is the outlet static pressure. It can be found that the pressure drop of the three types of corrugated plates shows a parabolic growth trend with speed. Among them, the double-hook corrugated plate has the fastest growth rate, followed by the single-hook corrugated plate. The growth rate of the non-hook corrugated plate is the slowest. The increase in inlet speed also leads to an increase in separation efficiency. The trend of increasing efficiency of the no-hook corrugated plate gradually becomes slower. There is a dividing point in the separation efficiency of single-hook and double-hook corrugated plates as the inlet speed increases. Therefore, the comparison of the separation efficiency of single-hook and double-hook corrugated plates needs to be combined with the inlet speed.
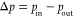
20

**Figure 6:**
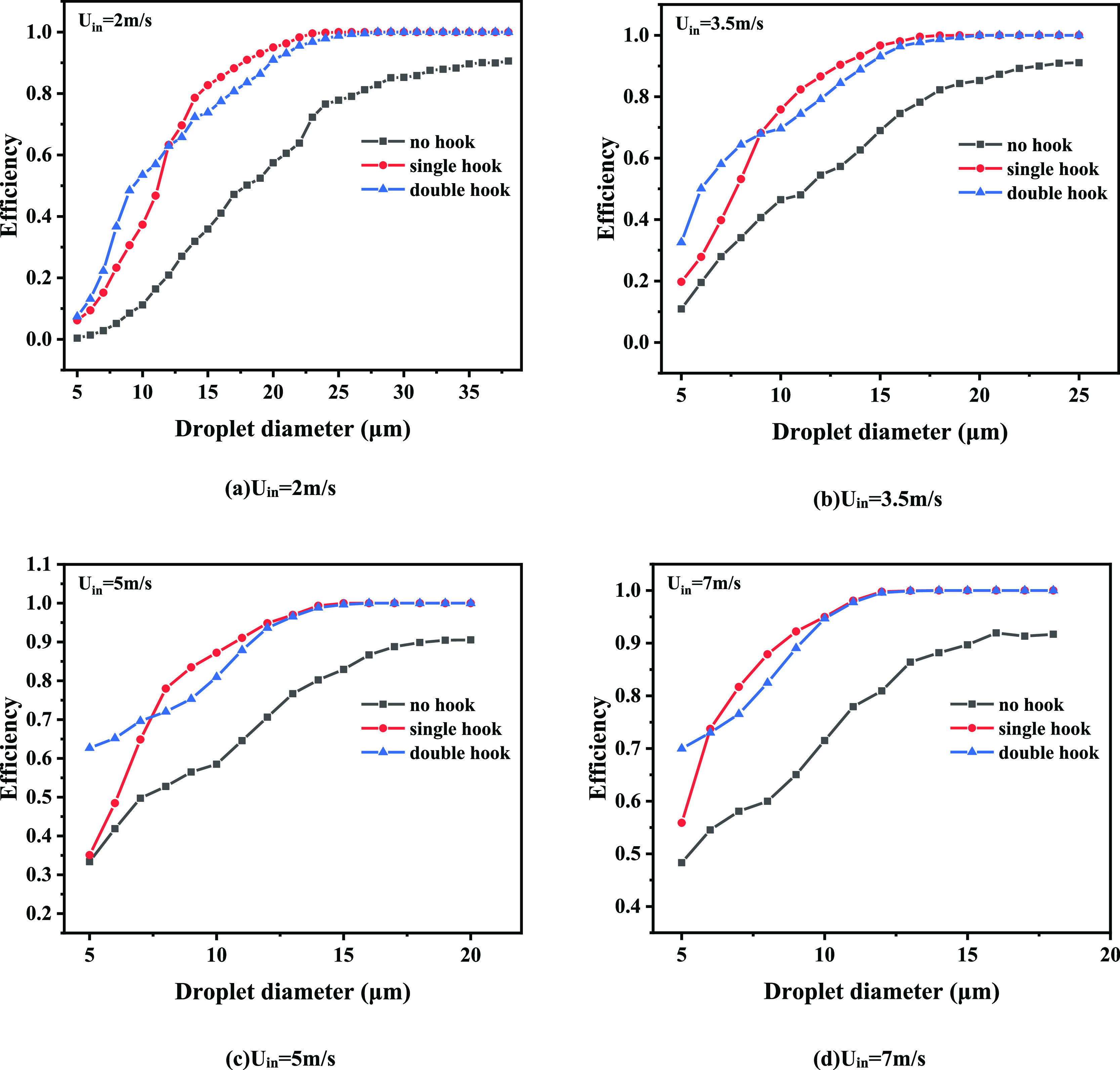
Variation of separation efficiency with speed: (a) *U*_in_ = 2 m/s, (b) *U*_in_ = 3.5 m/s, (c) *U*_in_ = 5 m/s, and (d) *U*_in_ = 7 m/s.

**Figure 7:**
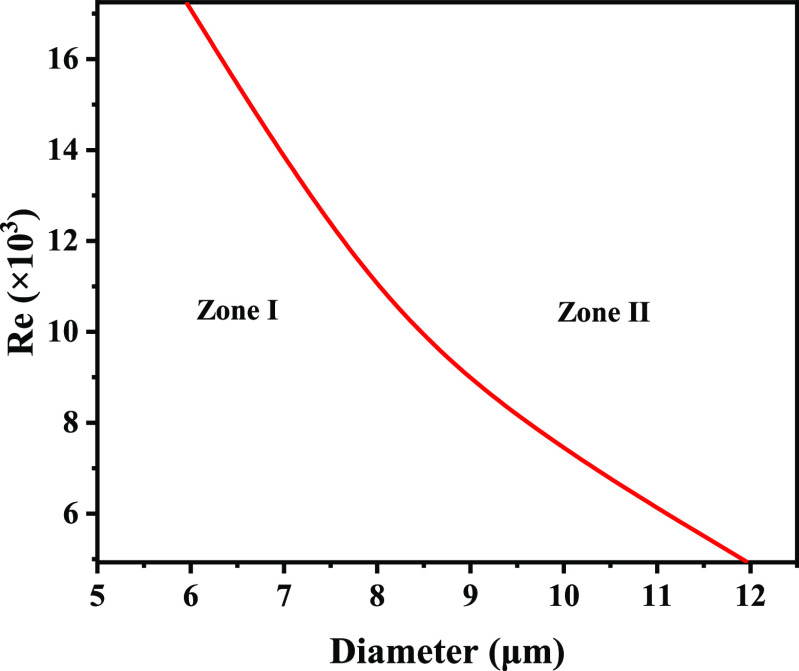
Advantageous diameter range for single- and double-hook corrugated plates.

As shown in Figure 6 and Table 3, the separation efficiency of the corrugated plate separator gradually increases with the increase of droplet diameter and finally reaches the limit value. The maximum value of separation efficiency of the non-hook corrugated plate is around 90%. For different sizes of droplets, the separation efficiency of the corrugated plate with hooks is always higher than that of the non-hook corrugated plate, and the separation efficiency eventually reaches 100% as the droplet diameter increases. The separation efficiency of single- and double-hook corrugated plates at different velocities is related to the droplet diameter. The droplet diameter at the same efficiency of the single-hook and double-hook corrugated plate is called the demarcation diameter, as shown in Table 3. Before the demarcation diameter, the double-hook corrugated plate has a higher separation efficiency. After the demarcation diameter, the separation efficiency of the single-hook corrugated plate is higher. As the velocity increases, the demarcation diameter decreases. Figure 7 is obtained according to the demarcation diameter at different velocities. The Reynolds number is introduced to indicate the effect of velocity. At the same *Re*, the separation efficiency of the double-hook corrugated plate is higher for droplets in the diameter range of zone I; for droplets in the diameter range of zone II, the separation efficiency of single-hook corrugated plates is higher. When separating droplets of a certain diameter, the double-hook corrugated plate has a higher separation efficiency when *Re* is in zone I. Conversely, the separation efficiency of the single-hook corrugated plate is higher.

**Table 3:** Maximum Efficiency η_max_ and Corresponding Droplet Diameter *d*_max_ at Different Speeds

velocity	2 m/s	3.5 m/s	5 m/s	7 m/s
η_max_–*d*_max_(no-hook)	100%–28 μm	100%–21 μm	100%–16 μm	100%–14 μm
η_max_–*d*_max_(single-hook)	100%–25 μm	100%–19 μm	100%–15 μm	100%–14 μm
η_max_–*d*_max_(double-hook)	90.57%–38 μm	91.07%–25 μm	90.53%–20 μm	91.96%–16 μm
efficiency demarcation diameter (single-hook and double-hook)	11.97 μm	8.97 μm	7.45 μm	5.95 μm

### 3.2 Effect of Plate Hook Spacing

In this paper, the effect of plate hook spacing on the separation performance of the corrugated plate with hooks is studied. The combination of plate hook spacing and channel width is dimensionless, and the ratio of plate hook spacing to channel area is expressed by *K*_a_, which is calculated as
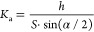


**Figure 8:**
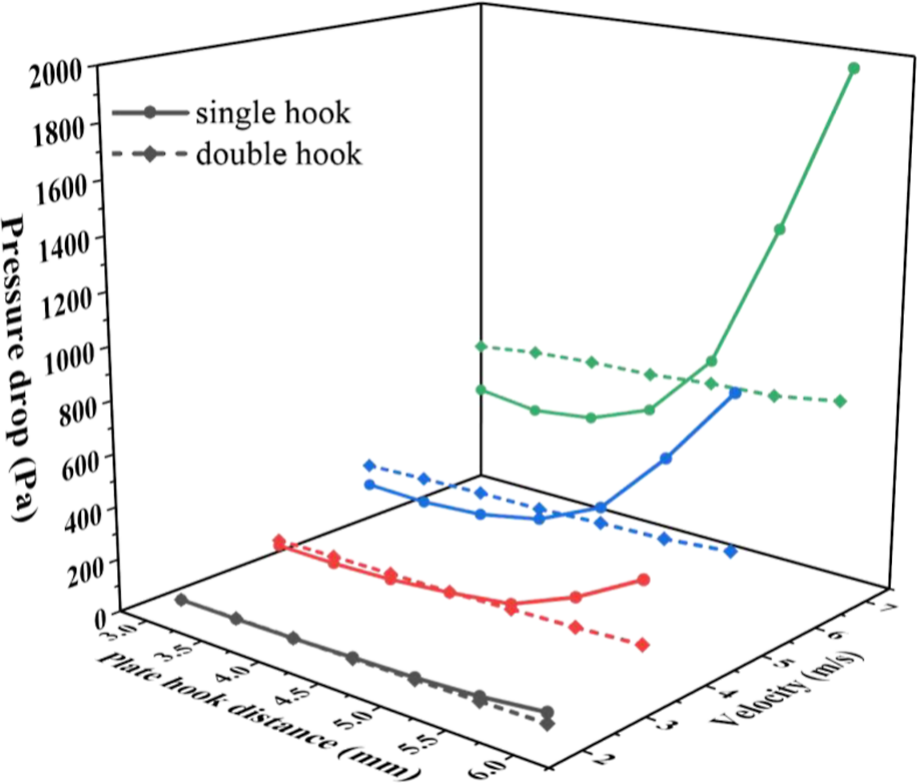
Effect of plate hook spacing on pressure drop.

As shown in Figure 8, the pressure drop of the single-hook corrugated plate and double-hook corrugated plate increases with the increase of the plate-hook distance at different speeds. This is because the increase in the plate-hook distance causes the corrugated plate internal flow channel area to decrease, the local velocity to increase, and the local resistance loss to increase. At different velocities, the pressure drop of single-hook and double-hook corrugated plates is related to the plate-hook distance. The plate hook distance when the pressure drops of single-hook and double-hook corrugated plates are the same is called the pressure drop demarcation plate–hook distance. Before the pressure drop demarcation plate–hook distance, the pressure drop of the double-hook corrugated plate is higher. After the pressure drop demarcation plate–hook distance, the pressure drop of the single-hook corrugated plate is higher. As the velocity increases, the pressure drop demarcation plate–hook distance gradually becomes larger.

**Figure 9:**
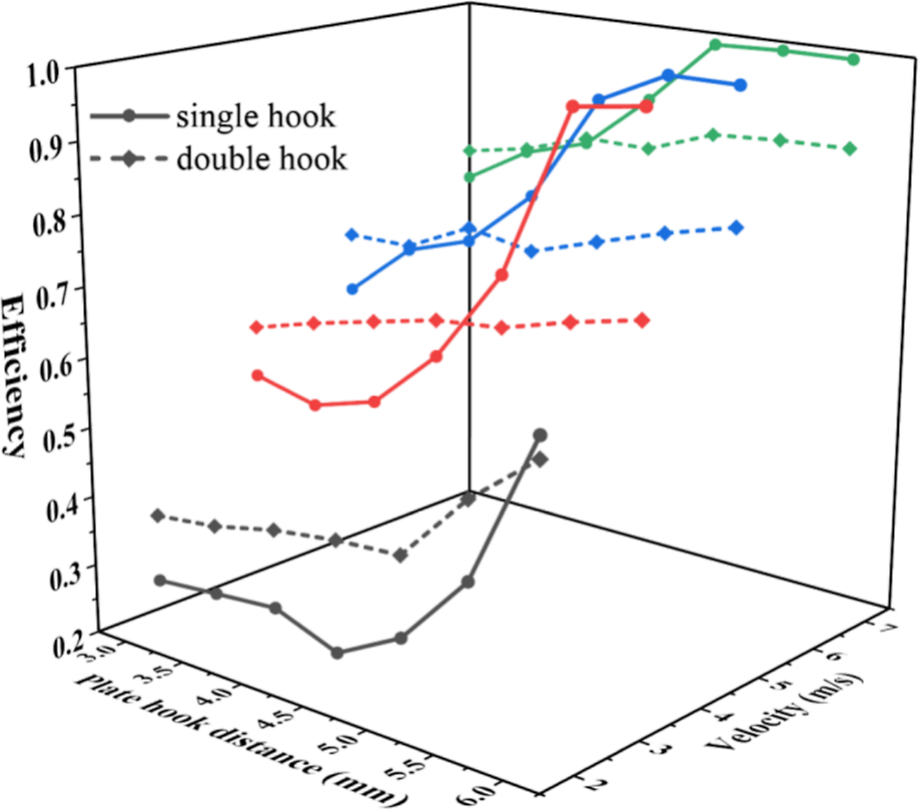
Effect of plate hook spacing on separation efficiency.

As shown in Figure 9, the separation efficiency of the corrugated plate increases with the increase of the plate hook distance at different velocities. This is because the drain hook occupies a larger flow channel area and the probability of droplets being trapped increases. At different velocities, the separation efficiency of single-hook and double-hook corrugated plates is related to the plate–hook distance. The plate–hook distance that has the same separation efficiency for single-hook and double-hook corrugated plates is called the efficiency demarcation plate–hook distance. Before the efficiency demarcation plate–hook distance, the separation efficiency of the double-hook corrugated plate is higher. After the efficiency demarcation plate–hook distance, the separation efficiency of the single-hook corrugated plate is higher. As the velocity increases, the efficiency demarcation plate–hook distance decreases.

**Figure 10:**
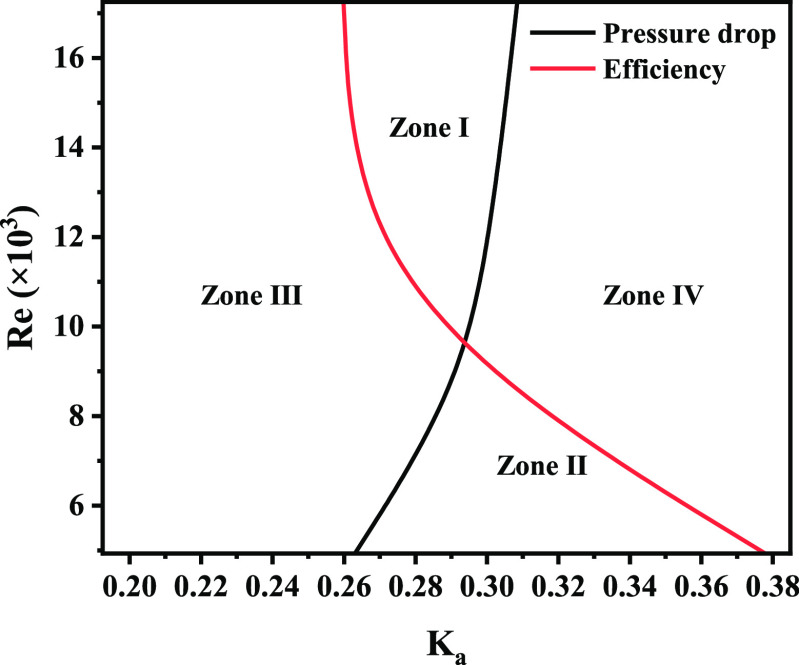
Single- and double-hook corrugated plate advantageous range.

The performance advantage interval of the single-hook corrugated plate and double-hook corrugated plate is obtained according to the demarcation plate–hook distance of pressure drop and efficiency. Figure 10 contains the efficiency divider and the pressure drop divider. On the left side of the efficiency dividing line, the separation efficiency of the double-hook corrugated plate is higher; conversely, the separation efficiency of the single-hook corrugated plate is higher. On the left side of the pressure drop dividing line, the pressure drop of the double-hook corrugated plate is higher; on the contrary, the pressure drop of the single-hook corrugated plate is higher. The efficiency dividing line intersects with the pressure drop dividing line to form four zones, and the characteristics of each zone are shown in Table 4.

**Table 4:** Single- and Double-Hook Corrugated Plate Performance Comparison

zone	η_sh_ vs η_dh_	Δ*P*_sh_ vs Δ*P*_dh_
zone I	>	<
zone II	<	>
zone III	<	<
zone IV	>	>

In zone I, the single-hook corrugated plate has a higher efficiency, a lower pressure drop, and better overall performance; in zone II, the double-hook corrugated plate has a higher efficiency, a lower pressure drop, and better overall performance. In actual application, the corrugated plate separator main configuration is usually determined to be several configurations. For a corrugated plate separator with a given plate–hook distance, the respective economic flow rate intervals for single-hook and double-hook corrugated plate separators are determined according to Figure 10. It is of great value for guiding industrial applications, reducing energy consumption, and improving economic efficiency.

### 3.3 Velocity Distribution and Droplet Motion Trajectory

#### 3.3.1 Velocity Distribution

**Figure 11:**
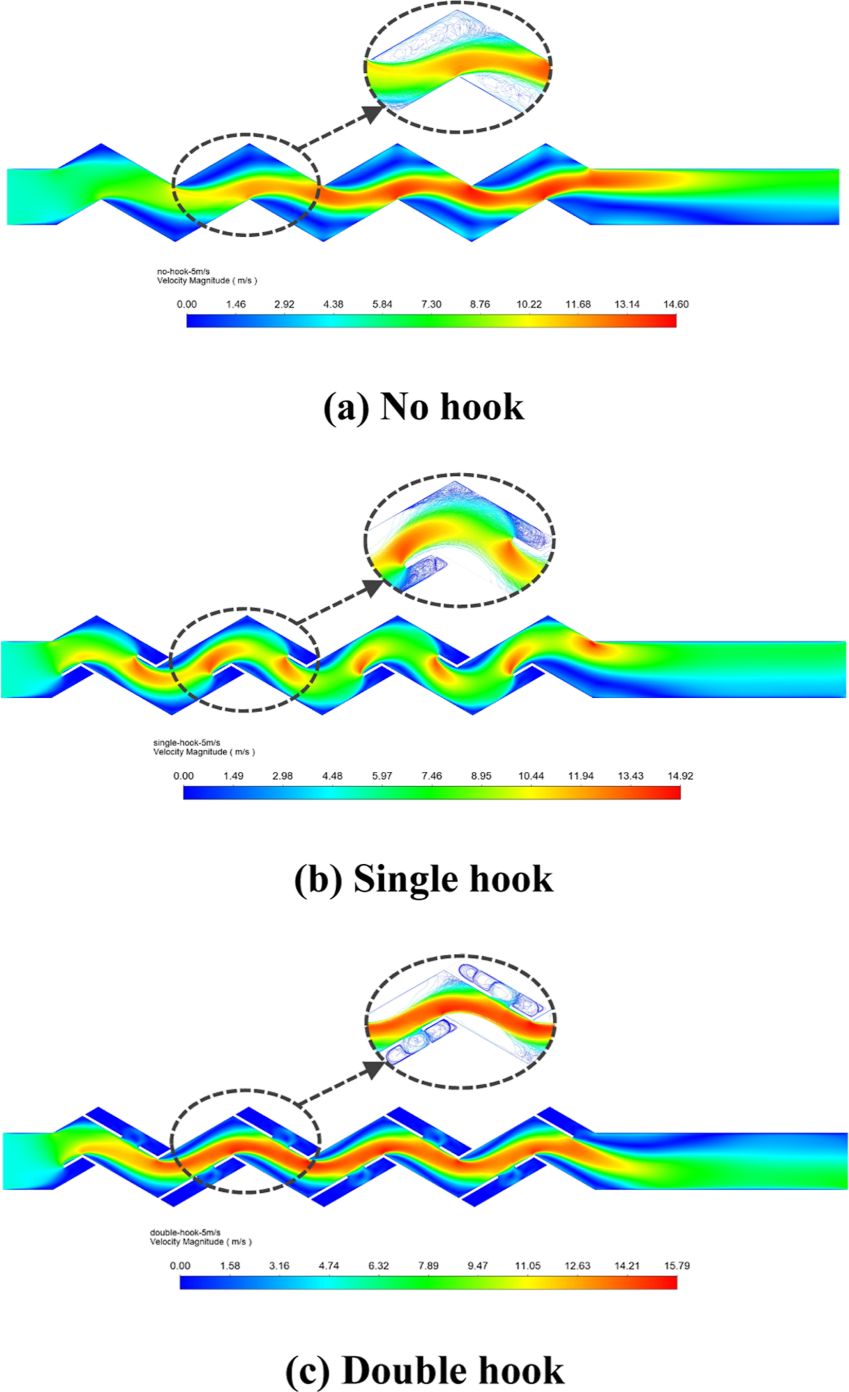
Velocity cloud diagram and local droplet motion trajectory: (a) no hook; (b) single hook; and (c) double hook.

Figure 11 shows the velocity distribution inside the corrugated plate of different configurations at the same velocity. From the figure, we can see that the presence of drainage hooks increases the local flow velocity. The maximum velocity of the corrugated plate with single hooks was at the front of the drainage hooks, and the maximum velocity of the corrugated plate with double hooks was at the bend of the rear drainage hooks. The corrugated plate with hooks has obvious swirls inside the drainage hooks.

**Figure 12:**
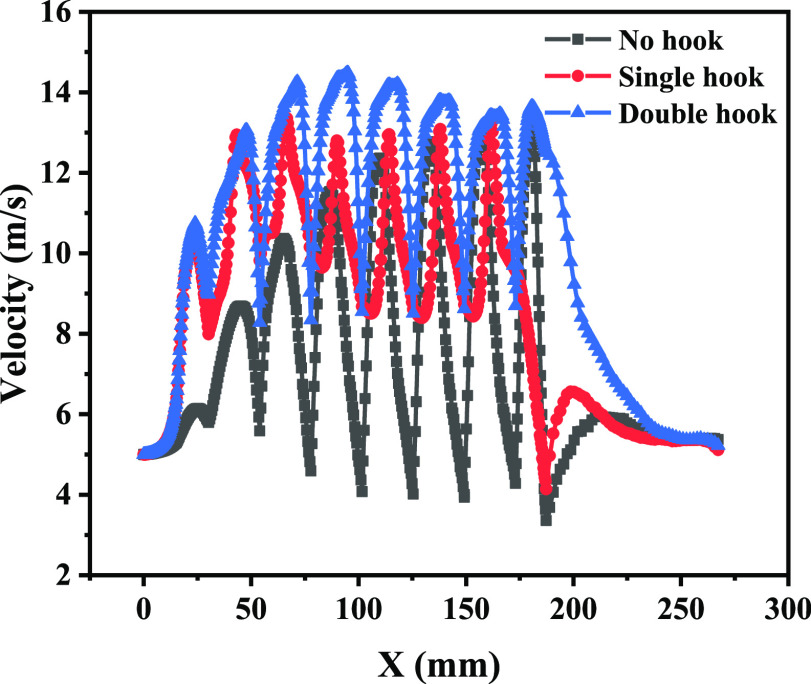
Variation of centerline velocity in the *x*-direction for different plate types.

**Figure 13:**
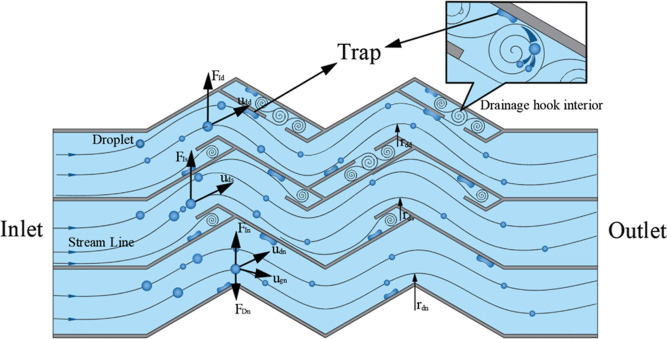
Schematic diagram of the force on a liquid drop.

The difference in density of the gas and liquid phases leads to different inertial forces. When the velocity changes strongly, the liquid phase cannot change its motion in time due to its large inertia, which is then trapped by the wall. Therefore, the frequent change in velocity is beneficial to gas–liquid separation. In order to further understand the change of velocity inside the corrugated plate, the velocity distribution of the centerline inside the corrugated plate of the three configurations was studied. According to Figure 12, the internal centerline velocity of the corrugated plate varies approximately periodically. Compared with that of the unhooked corrugated plate, the maximum velocities of the single-hooked and double-hooked corrugated plates increased by 2.19 and 8.15%, respectively. The gas velocity inside the corrugated plate fluctuated at a higher level with the aid of the drainage hooks, which enhanced the gas–liquid separation effect. According to the force analysis of the droplet motion in the corrugated plate by Nakao,^23^ as shown in Figure 13, the ratio of the inertial force and drag force *F*_I_/*F*_D_ is used to describe the effect of velocity.
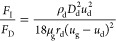
21where *r*_d_ is the radius of curvature of the droplet motion.

According to eq 21, it can be found that the larger the droplet diameter is, the more significant the inertia force effect is, the larger the droplet motion velocity is, the smaller the radius of curvature of the trajectory is, and the larger the *F*_I_/*F*_D_ ratio is, the easier the droplet is separated. The presence of the drain hook increases the local flow velocity at the zigzag corner, and at the same time, the physical blocking effect of the drain hook reduces the radius of curvature of the droplet motion, making the inertia force effect more obvious relative to that of the traction force, thus enhancing the separation effect.

#### 3.3.2 Droplet Motion Trajectory

**Figure 14:**
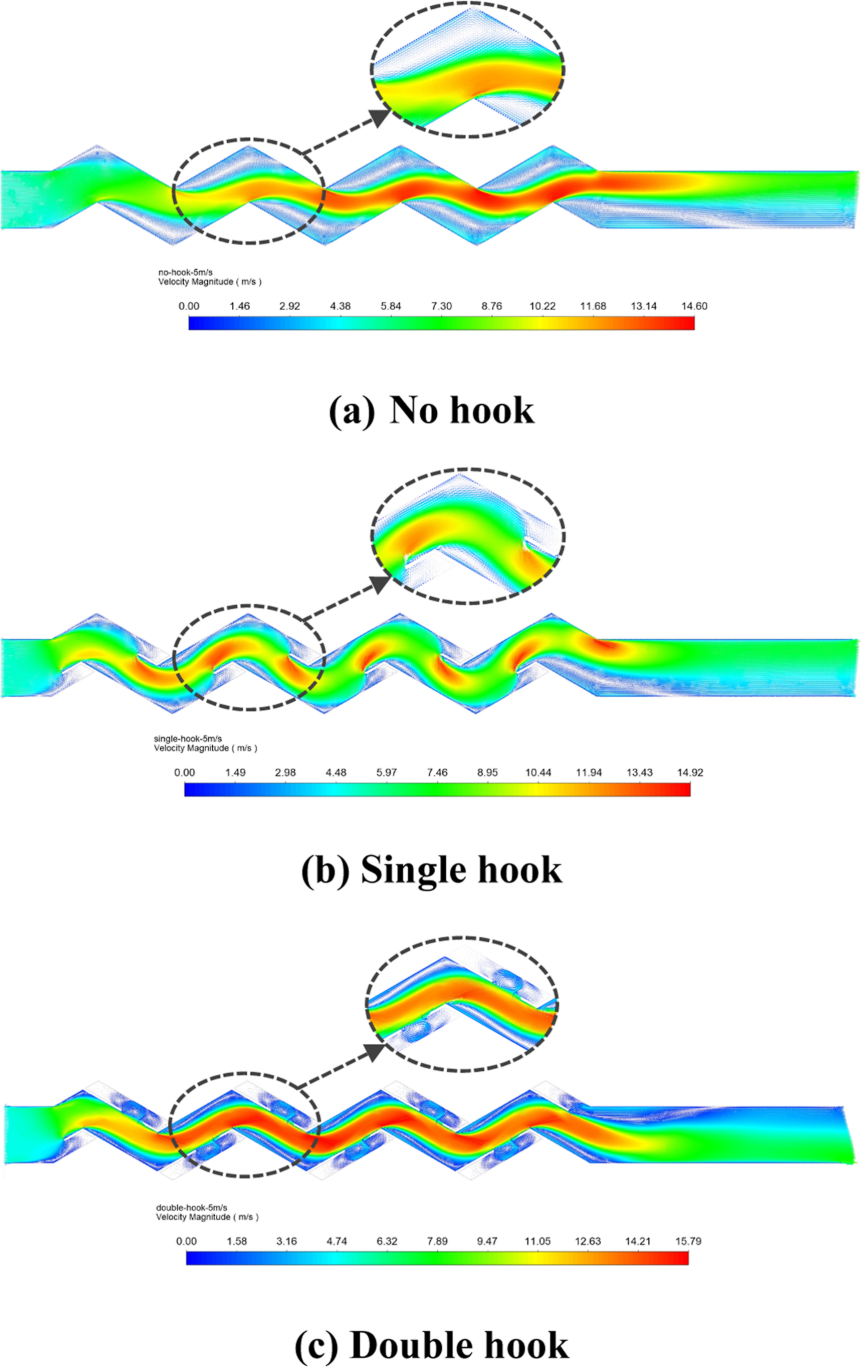
Velocity vector diagram of different plate types.

**Figure 15:**
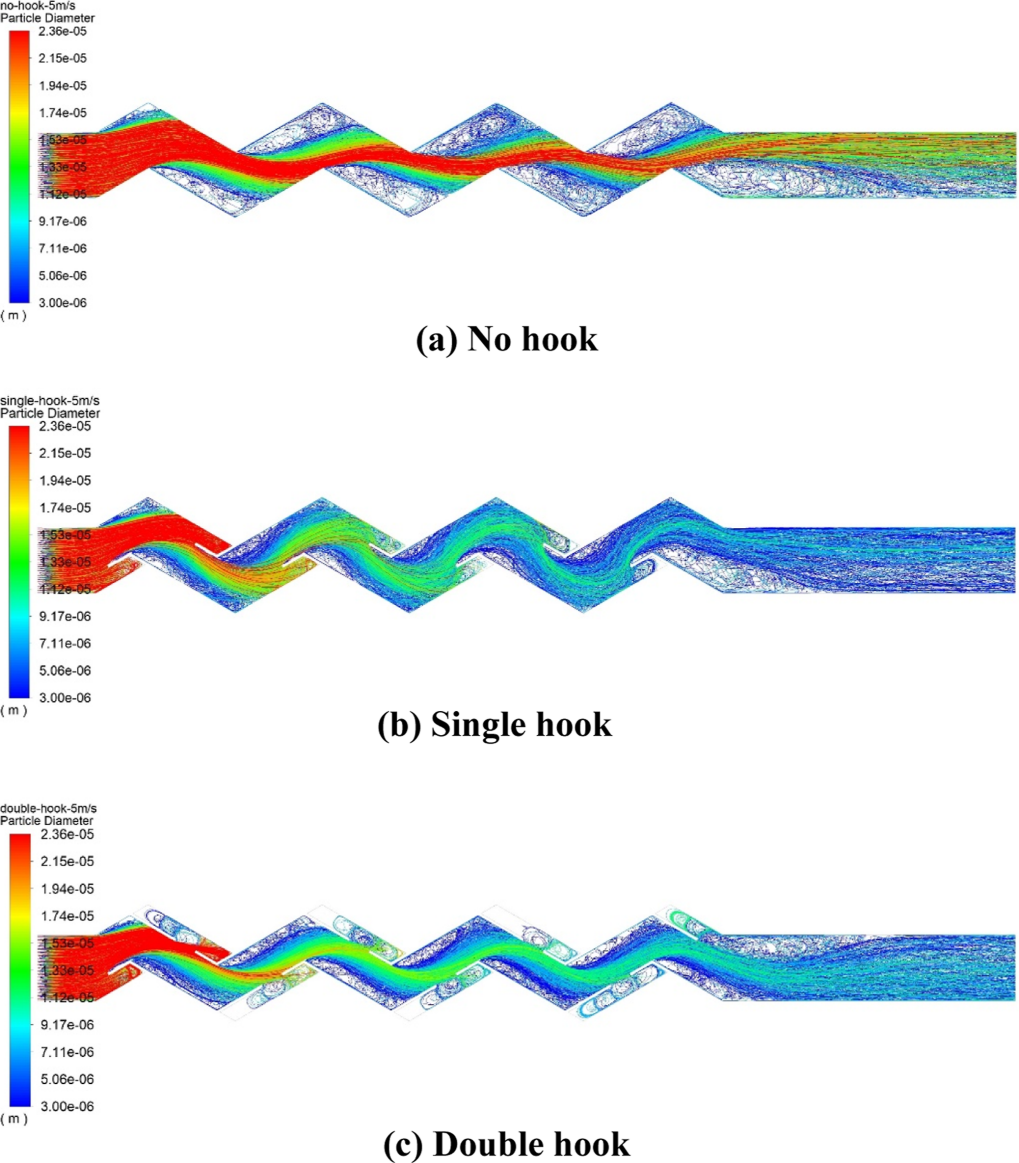
Trajectory of droplet movement of different plate types.

Figure 14 shows the velocity vector diagram of the airflow inside the corrugated plate. As we can see from the partial enlargement, an eddy is formed by the airflow inside the drainage hook. Combined with the droplet motion trajectory given in Figure 15, it can be found that a large number of droplets with small diameters are trapped in the eddy inside the drainage hook. According to the literature,^45^ if *L*_e_ > τ_d_(*u*_g_ – *u*_d_), the droplet will be trapped in the eddy; if *L*_e_ < τ_d_(*u*_g_ – *u*_d_), the droplet will escape from the eddy. According to eq 16, τ_d_ is related to the droplet diameter. In the eddy, small diameter droplets collide and cluster to form large diameter droplets. When the droplet diameter is large enough, it escapes from the eddy due to centrifugal force. At this moment, the drain hook blocks the droplets that escaped from the eddy from being re-entrained into the airflow, allowing them to be trapped on the wall. The drainage hooks improve the separation efficiency by creating an eddy and preventing secondary entrainment.

## 4 Conclusions

In this paper, the performance of different configurations of corrugated plates was compared by numerical simulation, the effect of plate hook spacing on pressure drop and separation efficiency was studied, and the principle of the enhanced effect of drainage hooks was analyzed, and the conclusions are as follows.

(1) With the assistance of drainage hooks, the separation efficiency of the corrugated plate with hooks is always higher than that of the corrugated plate without hooks. The maximum separation efficiencies are 90 and 100%, respectively.
(2) The separation efficiency of single- and double-hook corrugated plates is related to the droplet diameter. On the left side of the droplet diameter dividing line, the separation efficiency of the double-hook corrugated plate is higher; on the contrary, the separation efficiency of the single-hook corrugated plate is higher.
(3) The pressure drop and separation efficiency of single-hook and double-hook corrugated plates increase as the plate hook distance increases. The single-hook and double-hook corrugated plate performance is judged based on the efficiency demarcation plate hook distance and pressure drop plate–hook distance. When *Re* = 9.64 × 10^3^ and *K*_a_ = 0.294, the separation effects of single-hook and double-hook corrugated plates are the same.
(4) The draining hook’s assisted action mechanism is the formation of internal eddy and prevention of secondary entrainment.

## Abbreviations

*H*_1_: length of the inlet section (mm)
*H*_2_: length of the outlet section (mm)
*S*: plate angle (mm)
*h*: plate–hook spacing (mm)
λ: length of each stage (mm)
*u*: velocity (m·s^–1^)
*t*: time (s)
Reynolds stress tensor
*F*_D_: drag force per unit mass of particles (N)
*F*_I_: inertia force (N)
μ: dynamic viscosity (N s·m^–2^)
*g*: gravitational acceleration (m·s^–2^)
ρ: density (kg·m^–3^)
*k*: turbulence kinetic energy (m^2^·s^–2^)
ε: dissipation rate (m^2^·s^–3^)
*G*_*k*_: generation of *k* (kg·m^–1^ s^–2^)
*F*_*x*_: additional acceleration (N)
*Re*: relative Reynolds number
*C*_D_: drag force coefficient
*a*_1_, *a*_2_, *a*_3_: constants
*u̅*: continuous phase average velocity (m·s^–1^)
*u*′: fluctuation of continuous phase velocity (m·s^–1^)
*T*_L_: integral time scale (s)
*C*_L_: integral time scale constant
*y*^+^: dimensionless wall distance
τ_e_: eddy life time (s)
τ_d_: droplet relaxation time (s)
*L*_e_: Eddy scale
*t*_cross_: time for droplet crossing the eddy (s)
*m*_trap_: mass of trapped droplets (kg)
*m*_inject_: mass of injected droplets (kg)
*K*_a_: ratio of plate–hook spacing to channel width
*r*_d_: the radius of curvature of the droplet motion

## Subscripts

d: droplet
g: gas
*i*,*j*: indexes

## References

[ref1] Shi J.; Wang J.; Yu G. Chemical Industry Handbook, Chemical Industry Press, 1996.

[ref2] Wang B.; Zhang Z.; Li Q. Research and application of a new type of high efficient mesh mist eliminator. Mod. Chem. Ind. 2004, 5, 50–51.

[ref3] Dziubak T.; Bąkała L. Computational and Experimental Analysis of Axial Flow Cyclone Used for Intake Air Filtration in Internal Combustion Engines. Energies 2021, 14, 2285 10.3390/en14082285.

[ref4] Dziubak T. Experimental Investigation of Possibilities to Improve Filtration Efficiency of Tangential Inlet Return Cyclones by Modification of Their Design. Energies 2022, 15, 3871 10.3390/en15113871.

[ref5] Dziubak T. Experimental Studies of Dust Suction Irregularity from Multi-Cyclone Dust Collector of Two-Stage Air Filter. Energies 2021, 14, 3577 10.3390/en14123577.

[ref6] Bojdo N.; Filippone A. A Simple Model to Assess the Role of Dust Composition and Size on Deposition in Rotorcraft Engines. Aerospace 2019, 6, 44 10.3390/aerospace6040044.

[ref7] Kashani E.; Mohebbi A.; Heidari M. G. CFD simulation of the preheater cyclone of a cement plant and the optimization of its performance using a combination of the design of experiment and multi-gene genetic programming. Powder Technol. 2018, 327, 430–441. 10.1016/j.powtec.2017.12.091.

[ref8] Padovan L. In Design of an innovative moisture separator technology for use in nuclear power plants: Numerical approach — Part 1, International Conference on Nuclear Engineering, Proceedings, ICONE, 2020.

[ref9] Noh S.-Y.; Kim M.-W.; Yook S.-J. Performance Investigation of a Vertical Wave-plate Mist Eliminator with Perforated Plates. Aerosol Air Qual. Res. 2020, 20, 2681–2689. 10.4209/aaqr.2020.05.0191.

[ref10] Yu Z.; Sun C.; Fang J.; Zhang L.; Hu Y.; Bao B.; Bu S.; Xu W.; Ji Y. Water recovery efficiency improvement using the enhanced structure of the mist eliminator. Process Saf. Environ. Prot. 2021, 154, 433–446. 10.1016/j.psep.2021.08.018.

[ref11] Koopman H. K.; Köksoy Ç.; Ertunç Ö.; Lienhart H.; Hedwig H.; Delgado A. An analytical model for droplet separation in vane separators and measurements of grade efficiency and pressure drop. Nucl. Eng. Des. 2014, 276, 98–106. 10.1016/j.nucengdes.2014.05.034.

[ref12] Fang C.; Zou R.; Luo G.; Ji Q.; Sun R.; Hu H.; Li X.; Yao H. CFD simulation design and optimization of a novel zigzag wave-plate mist eliminator with perforated plate. Appl. Therm. Eng. 2021, 184, 116212 10.1016/j.applthermaleng.2020.116212.

[ref13] Hamedi Estakhrsar M. H.; Rafee R. Effects of wavelength and number of bends on the performance of zigzag demisters with drainage channels. Appl. Math. Model. 2016, 40, 685–699. 10.1016/j.apm.2015.08.023.

[ref14] James P. W.; Wang Y.; Azzopardi B. J.; Hughes J. P. The Role of Drainage Channels in the Performance of Wave-Plate Mist Eliminators. Chem. Eng. Res. Des. 2003, 81, 639–648. 10.1205/026387603322150499.

[ref15] Wang X.; Huang S. Study of corrugated plates separator with single scoop. J. Huazhong Univ. Sci. Technol. (Nat. Sci. Ed.) 2004, 32, 63–65.

[ref16] Wang X.; Huang S. A experimental research on the new and high efficiency steam-water separator. J. Eng. Thermophys. 2005, 26, 97–100.

[ref17] Li J.; Huang S.; Wang X. Experimental Research of Separation Efficiency on Steam-Water Separator with Corrugated Plates. Nucl. Power Eng. 2007, 28, 94–97.

[ref18] Li J.; Huang S.; Wang X.; Kuang J. Experimental research of cold state operation of corrugated-plate separator. J. Huazhong Univ. Sci. Technol. (Nat. Sci. Ed.) 2008, 36, 112.

[ref19] Li J.; Huang S.; Wang X. Analysis of the Influence of Turbulent Flow Effect on the Corrugated Plate Stream-Water Separators With Drain Hooks. Chem. Eng. Mach. 2008, 36, 282–286.

[ref20] Li J.; Huang S.; Wang X. Analysis and Computation for Corrugated Plates Dryer Considering Reentrainment. Nucl. Power Eng. 2009, 30, 100–103.

[ref21] Chen J.; Xue Y.; Wang X.; Chen H.; Liu H.; Ba C.; Zuo C. Proof Test in Hot Condition on Steam Separation Device in Steam Generator for 1000MW PWR Nuclear Power Plant. Nucl. Power Eng. 2006, 27, 61–66.

[ref22] Chen J.; Cheng H.; Xue Y.; Wang X.; Liu H.; Ba C.; Zuo C. Test and Study of Cold Condition for Dryer in Steam Generator for 1000MW PWR Nuclear Power Plant. Nucl. Power Eng. 2006, 27, 72–77.

[ref23] Nakao T.; Saito Y.; Souma H.; Kawasaki T.; Aoyama G. Droplet Behavior Analyses in the BWR Dryer and Separator. J. Nucl. Sci. Technol. 1998, 35, 286–293. 10.1080/18811248.1998.9733858.

[ref24] Nakao T.; Nagase M.; Aoyama G.; Murase M. Development of Simplified Wave-type Vane in BWR Steam Dryer and Assessment of Vane Droplet Removal Characteristics. J. Nucl. Sci. Technol. 2012, 36, 424–432. 10.1080/18811248.1999.9726225.

[ref25] Jøsang A. I.; Melaaen M. C. Fluid Flow Simulations of a Vane Separator. Modeling, Identification and Control: A Norwegian Research Bulletin 2002, 23, 5–26. 10.4173/mic.2002.1.1.

[ref26] Galletti C.; Brunazzi E.; Tognotti L. A numerical model for gas flow and droplet motion in wave-plate mist eliminators with drainage channels. Chem. Eng. Sci. 2008, 63, 5639–5652. 10.1016/j.ces.2008.08.013.

[ref27] Rafee R.; Rahimzadeh H.; Ahmadi G. Numerical simulations of airflow and droplet transport in a wave-plate mist eliminator. Chem. Eng. Res. Des. 2010, 88, 1393–1404. 10.1016/j.cherd.2010.03.001.

[ref28] Makowski Ł.; Łaskowski J.; Tyrański M. Influence of modification of the geometry of the wave-plate mist eliminators on the droplet removal efficiency— cfd modelling. Processes 2021, 9, 1499 10.3390/pr9091499.

[ref29] Kavousi F.; Behjat Y.; Shahhosseini S. Optimal design of drainage channel geometry parameters in vane demister liquid–gas separators. Chem. Eng. Res. Des. 2013, 91, 1212–1222. 10.1016/j.cherd.2013.01.012.

[ref30] Liang Q.; Mao J.; Wang F.; He Y. Optimization of Structural Parameters for Corrugated Plates Separator With Single Scoop. J. Eng. Thermophys. 2016, 37, 796–802.

[ref31] Artemov V.; Minko K.; Yankov G.; Ptakhin A.; Kondratev A.; Milman O. Numerical simulation of gas flow and droplet motion in a wave-plate eliminator of the separator-steam-generator system in the waste-heat-utilisation complex. EPJ Web Conferences, 2017.

[ref32] Zhang H.; Bo H. L. Numerical Study on Separation Ability and Structure Optimization of AP1000 Secondary Steam-water Separator. At. Energy Sci. Technol. 2014, 48, 185.

[ref33] Wang P.; Jiang J.; Li S.; Yang X.; Luo X.; Wang Y.; Thakur A. K.; Zhao W. Numerical investigation on the fluid droplet separation performance of corrugated plate gas-liquid separators. Sep. Purif. Technol. 2020, 248, 117027 10.1016/j.seppur.2020.117027.

[ref34] Li S.; Wang P.; Luo X.; Wang Y.; Yang X.; Thakur A. K.; Zhao W. Numerical analysis of chevron demisters with drainage hooks in optimizing separation performance. Int. J. Heat Mass Transfer 2020, 152, 119522 10.1016/j.ijheatmasstransfer.2020.119522.

[ref35] Ma W.; Wu X.; Ji Z. Separation performance optimization of wave-plate mist eliminator based on response surface methodology. Chin. J. Process Eng. 2018, 18, 689–696. 10.12034/j.issn.1009-606X.217369.

[ref36] Xu Y.; Yang Z.; Zhang J. Study on performance of wave-plate mist eliminator with porous foam layer as enhanced structure. Part I: Numerical simulation. Chem. Eng. Sci. 2017, 171, 650–661. 10.1016/j.ces.2017.05.031.

[ref37] Zamora B.; Kaiser A. S. Comparative efficiency evaluations of four types of cooling tower drift eliminator, by numerical investigation. Chem. Eng. Sci. 2011, 66, 1232–1245. 10.1016/j.ces.2010.12.023.

[ref38] Tang Y.; Xu Y.; Zhang B.; He C.; Chen Q.; Ren J. An integrated computational strategy for the geometric design and prioritization of wave-plate mist eliminators. Process Saf. Environ. Prot. 2022, 158, 674–686. 10.1016/j.psep.2021.12.039.

[ref39] Venkatesan G.; Kulasekharan N.; Iniyan S. Influence of turbulence models on the performance prediction of flow through curved vane demisters. Desalination 2013, 329, 19–28. 10.1016/j.desal.2013.09.001.

[ref40] Venkatesan G.; Kulasekharan N.; Iniyan S. Numerical analysis of curved vane demisters in estimating water droplet separation efficiency. Desalination 2014, 339, 40–53. 10.1016/j.desal.2014.02.013.

[ref41] ANSYS FLUENT 14.5. User’s and theory guide, 2014.

[ref42] Morsi S. A.; Alexander A. J. An investigation of particle trajectories in two-phase flow systems. J. Fluid Mech. 1972, 55, 193 10.1017/s0022112072001806.

[ref43] Estakhrsar M. H. H.; Rafee R. Effect of drainage channel dimensions on the performance of wave-plate mist eliminators. Korean J. Chem. Eng. 2013, 30, 1301–1311. 10.1007/s11814-013-0032-9.

[ref44] Ghetti S. Investigation of entrainment phenomena in inertial separators. M.S. Thesis, University of Pisa, Pisa, Italy, 2003.

[ref45] Zhao C. The Numerical Simulation and Optimization of the Wave-Plate Mist Eliminator with Auxiliary Capture; Tianjin University: Tinajin, China, 2015.

